# Continued Need for Comprehensive Genetic and Phenotypic Characterization of Viruses: Benefits of Complementing Sequence Analyses with Functional Determinations

**DOI:** 10.4269/ajtmh.18-0128

**Published:** 2018-03-19

**Authors:** Aaron C. Brault, Bradley J. Blitvich

**Affiliations:** 1 Division of Vector-Borne Diseases, Centers for Disease Control and Prevention, Fort Collins, Colorado;; 2 Department of Veterinary Microbiology and Preventive Medicine, College of Veterinary Medicine, Iowa State University, Ames, Iowa

The expanded use of next generation sequencing tools has led to an explosion in the rate of discovery of novel viral agents and has had a measured effect on the capacity to genetically identify the presence of previously described viruses in new geographic environments and within different hosts and vectors. For example, more than 30 flaviviruses (*Flavivirus*; *Flaviviridae*) with the capacity to infect only mosquitoes have been described in the last 10 years. By contrast, only two such viruses had been described in the previous 33 years.^1^ This burgeoning expansion of the known virome has largely outpaced the scientific capacity for characterizing these agents in any detail. Furthermore, many of the methodologies used for rapid genetic detection (such as placing mosquitoes directly in nucleic acid extraction buffers and the use of FTA cards for blood samples) preclude the isolation of the agents.

A slowed pace of arbovirus discovery in international tropical locations (1970–2005) has been associated with the cessation of directed virus discovery efforts by the Rockefeller Foundation in the late 1960s.^2^ However, the aforementioned technological developments that have precluded isolation of viruses have been conducive for detection of these agents in remote areas and have been more readily applied because of the reduced need to establish laboratory facilities in remote locations and maintain a cold chain. The need for field-based laboratories has largely been replaced by field collections with the subsequent shipment of samples to established laboratories for sequencing at alternative locations. As such, the use of sequence data and genetic relatedness between viruses has become the new standard for taxonomic classification of novel agents and determining the distribution of known agents.^3^ Taxonomic assignments have further often been made without an actual isolate of the agent. Although molecular studies provide considerable information on the distribution, host/vector associations, and evolutionary relationships between and selection pressures acting on different viruses, the absence of actual cultured isolates has impeded the phenotypic or serological characterization that has historically provided important information on the complete host/vector ranges (through competence testing in alternative vector and/or hosts), disease potential (through pathogenesis testing), and potential for serological protection against known human disease agents.

In this issue of the *American Journal of Tropical Medicine and Hygiene*, a collaborative group of investigators from Brazil and the United States genetically and phenotypically describe viruses from the Gamboa serocomplex (*Orthobunyavirus*; Peribunyaviridae).^4^ The investigators identified four genotypes within the serocomplex, evidence of reassortment events among the three genomic segments, and the existence of more complicated genetic relationships between the viruses that were previously identified by classical serological techniques (complement fixation and neutralization assays). The study demonstrates the power of combining archival and prospective sampling for viruses in concert with new technologies that allow for the rapid genetic characterization of viruses. The existence of isolates allowed the investigators to determine the seroprevalence of Gamboa virus in birds and mammals, and it was revealed that exposure rates are considerably higher in birds. Coupled with the isolation of viruses from birds and ornithophilic mosquitoes, these data further implicate birds as important reservoir hosts. Pathological characterization in newborn chickens demonstrated seroconversion with limited disease presentation. Although no human disease association with Gamboa virus has been identified, these kinds of studies provide a comprehensive presentation of genetic, antigenic, and epidemiological data from which a more complete appreciation for human and veterinary disease potential can be assessed, and serve as a reminder that without balanced efforts to produce material from field samples that can be used for phenotypic and serological characterization, only a partial understanding of viral epidemiology and pathogenesis can be achieved.
